# Did the call for boycott by the Catholic bishops affect the polio vaccination coverage in Kenya in 2015? A cross-sectional study

**DOI:** 10.11604/pamj.2016.24.120.8986

**Published:** 2016-06-07

**Authors:** Ian Njeru, Yusuf Ajack, Charles Muitherero, Dickens Onyango, Johnny Musyoka, Iheoma Onuekusi, Jackson Kioko, Nicholas Muraguri, Robert Davis

**Affiliations:** 1 Ministry of Health, Nairobi, Kenya; 2 World Health Organization, Nairobi, Kenya; 3 American Red Cross, Nairobi, Kenya

**Keywords:** Polio vaccine, vaccination, vaccine safety, boycott, Catholic bishops

## Abstract

**Introduction:**

Polio eradication is now feasible after removal of Nigeria from the list of endemic countries and global reduction of cases of wild polio virus in 2015 by more than 80%. However, all countries must remain focused to achieve eradication. In August 2015, the Catholic bishops in Kenya called for boycott of a polio vaccination campaign citing safety concerns with the polio vaccine. We conducted a survey to establish if the coverage was affected by the boycott.

**Methods:**

A cross sectional survey was conducted in all the 32 counties that participated in the campaign. A total of 90,157 children and 37,732 parents/guardians were sampled to determine the vaccination coverage and reasons for missed vaccination.

**Results:**

The national vaccination coverage was 93% compared to 94% in the November 2014 campaign. The proportion of parents/guardians that belonged to Catholic Church was 31% compared to 7% of the children who were missed. Reasons for missed vaccination included house not being visited (44%), children not being at home at time of visit (38%), refusal by parents (12%), children being as leep (1%), and various other reasons (5%). Compared to the November 2014 campaign, the proportion of children who were not vaccinated due to parent's refusal significantly increased from 6% to 12% in August 2015.

**Conclusion:**

The call for boycott did not affect the campaign significantly. However, if the call for boycott is repeated in future it could have some significant negative implication to polio eradication. It is therefore important to ensure that any vaccine safety issues are addressed accordingly.

## Introduction

The World Health Organization (WHO) launched the Global Polio Eradication Initiative (GPEI) in 1988 with the goal of eradicating polio by the year 2000 [1]. Subsequently, a plan of action for global polio eradication was developed and approved in 1989 by the World Health Assembly and adopted by the African region [2]. This plan proposed four approaches to polio eradication: maintaining high routine immunization coverage, National Immunization Days (NIDs), Supplementary Immunization Activities (SIA) and surveillance for Acute Flaccid Paralysis (AFP). In 1996, the “Kick Polio out of Africa” was launched in Africa and targeted to vaccinate 50 million children within the year [3, 4]. Before the discovery of vaccines in 1955, polio used to paralyze or kill half a million people every year [5]. By 1988, polio was endemic in 125 countries paralyzing 350,000 children annually [6]. Although the goal to eradicate polio by the year 2000 was not met as planned, the global polio eradication initiative has made tremendous progress. Firstly, the transmission of wild polio virus has been interrupted in all countries except two; Pakistan and Afghanistan [6]. Secondly, the number of polio cases declined to only 359 in 2014 which represents greater than 99% reduction since 1988. Compared to 2014, the cases in 2015 declined further by approximately 80% [7]. Thirdly, neither type 2 nor type 3 virus has been detected recently [8]. Kenya has not been left behind in the war against polio and has made good progress in controlling the disease. The last indigenous polio case was detected in 1984. However, four outbreaks occurred in 2006, 2009, 2011 and 2013 as a result of importation from neighboring countries resulting in 2, 19, 1 and 14 wild polio cases respectively [7, 9]. The latest outbreak that occurred in 2013 was successfully controlled but the country remains at risk due to close proximity to other vulnerable countries, presence of refugee camps in the country and areas with sub optimal population immunity. In an effort to finally eradicate polio globally by 2018, the GPEI in consultation with national health authorities developed the “Polio Eradication and Endgame Strategic Plan 2013-2018” [10]. The plan has four objectives: detection and interruption of wild virus, strengthening routine immunization and withdrawal of oral polio vaccine, containment and certification and finally legacy planning. Under the first objective, outbreak response continues through the period of the strategy and this is implemented through immunization campaigns. The second objective has introduced certain milestones in which all countries were expected to introduce at least one dose of inactivated polio vaccine (IPV) into their routine immunization schedule by end of 2015. If the strategic plan is implemented well as planned, then polio eradication will become a reality. However, political and social barriers, including religious resistance to vaccination are threats to the success of the endgame strategy [11].

Generally, most religious groups have issued statements supportive of immunization. However, there has been some opposition to vaccination by religious groups due to various reasons. Some churches believe in healing everything through prayer rather than the administration of health products whose contents they are suspicious of [12]. According to a review by Grabenstein, causes of religious opposition can be categorized into three: violation of dietary laws, interference with natural order by not letting events take their course and violation of prohibitions against taking life [13]. Regardless of the cause, religious opposition to vaccination has the potential to derail the control of vaccine preventable diseases. As a matter of fact, numerous outbreaks of such diseases have occurred among non-vaccinated religious groups [14–18]. Religious opposition to polio vaccination has been associated with failure of immunization programs in Afghanistan, Pakistan and Nigeria [19–21]. In all three countries, Muslim fundamentalists objected to polio vaccination thereby derailing eradication efforts. In both Afghanistan and Pakistan, the Taliban issued edicts banning polio vaccination since they suspect that the vaccine contains a contraceptive [22]. In Nigeria three states boycotted a polio campaign conducted in August 2003. This was caused by a boycott order from the Supreme Council for Sharia on the suspicion that the vaccine contained contraceptives and Human Immuno-deficiency Virus (HIV) [21, 23, 24]. The boycott resulted in a resurgence of cases and wild polio virus outbreaks occurred in many African countries which had previously been disease frees [21]. Although polio campaigns in Kenya have previously been successful, religious resistance to mass vaccinations is beginning to take root. It all started in 2014 when the Catholic bishops complained that tetanus vaccine administered to women of reproductive age during a campaign was laced with contraceptives in the form of Human Chorionic Gonadotrophin (HCG) [25]. This claim was supported by the Kenya Catholic Doctors Association, a pro-vaccination group, which alleged that six samples of the tetanus vaccine tested positive for HCG [26, 27]. The government denied the claims and responded by appointing an independent committee to test the vaccine and the dispute fizzled out subsequently [28]. In January 2015, the Ministry of Health and the World Health Organization (WHO) conducted a polio risk analysis that identified 32(68%) counties to be at moderate to high risk of polio in Kenya. Two preventive vaccination campaigns were therefore planned for April and May 2015 targeting 32 and 11 counties, respectively. However, the Catholic bishops objected to the campaigns and demanded that the vaccines be tested first [29]. The campaigns were therefore postponed to August and September 2015 in order to allow for more time for consultation with the Catholic bishops. The government engaged the bishops and formed a committee to address the issues raised. The government also consulted other stakeholders who recommended that the campaigns should go on as planned since all the issues raised had been addressed. However, the Catholic bishops were not fully satisfied with the process and hence called on all their faithful to boycott the August 2015 polio campaign [30]. The population of the Catholic faithful in Kenya is 23% and therefore comprises a sizeable proportion of the population [31]. Boycotting the vaccination campaign has serious implications for the polio eradication agenda in Kenya and may reverse the gains made in the last few decades. We therefore conducted a survey immediately after the August 2015 campaign in order to find out if the call for boycott by the Catholic bishops did affect the polio vaccination coverage. The survey also aimed at determining the reasons for non vaccination. This paper presents the results of this survey.

## Methods

### Study design

This was a cross-sectional study that was conducted for 2 days following the polio campaign that was conducted in 32 counties from 1st to 5th August 2015. The findings of this study was compared to those of another cross-sectional study that was conducted in November 2014 following a similar polio vaccination campaign that was conducted on 8th to 12th November 2014 in all the 47 counties.

### Study participants

The eligible study participants for vaccination coverage were all the 6 million children less than 5 years that were targeted for the polio mass vaccination campaign in 32 of the 47 counties of Kenya. All the parents and guardians of the targeted children were also eligible for answering the questions on reasons for non vaccination. The 32 counties that participated in the vaccination campaign were selected based on a polio risk analysis tool that looks at data on population immunity, surveillance and population risk such as proximity to areas with a polio outbreak or presence of high risk groups such as refugee camps.

### Sampling procedure

The sampling to estimate the vaccination coverage following the mass vaccination was done in line with the recommendations of the Kenya Ministry of Health and the World Health Organization (WHO). The requirements are that at least 2% of the eligible children are sampled in at least 50% of all sub counties (districts) where the campaign is done. To ensure independence of the exercise, external assessors were hired and only children whose finger had been marked with indelible ink were considered as having been vaccinated. For this survey, multistage sampling was done all the way from the national level to the village and household level. The first stage of sampling was done at the national level where 100 (54%) of the 185 sub counties that participated in the campaign were randomly selected. This sampling was done in such a way as to ensure that all the 32 counties had at least one sub county selected for the exercise. In each county, sub counties which had unique challenges such as hard to reach communities, vastness and previous data quality issues were prioritized. From the 100 selected sub counties, the number of children to be sampled from each was allocated proportionate to size so as to achieve the minimum 2% expected nationally. The Sub county level was the second stage of sampling. Three divisions (1 urban, 2 rural) were selected from each sub county and one location was randomly selected from each division. Selection of divisions and locations was done randomly except in special areas with insecurity and low routine coverage where convenience and special interest was factored in. In each selected location, one sub location was randomly selected where 4 villages were randomly selected. In each village at least 20 households were sampled systematically to ensure even distribution. All children less than 5 years in the selected households were included in the study. In addition to the households sampled in the 4 villages per sub location, children were sampled in two public places per sub location such as schools, play grounds or churches. All children in the public places were sampled.

### Data collection and analysis

Data was collected using a short semi structured questionnaire that captured a number of indicators including number of eligible children, number of children with a finger mark(vaccinated), number of children without a finger mark (not vaccinated), reasons for non vaccination and religion of the head of the household. Data was transmitted to the national level for analysis using an electronic tool that had been captured in Google drive. Data analysis was done using Excel and Epi Info version 7 and included proportions of children vaccinated, proportions of children not vaccinated, and reasons for non vaccination, among others. Chi square test was used to establish if there was any statistically significant difference in coverage between the November 2014 campaign and the August 2015 campaign.

### Ethical approval

The study was approved by the Ministry of Health as part of routine post polio campaign coverage survey and was therefore exempted from ethical committee review.

## Results

**Figure 1:**
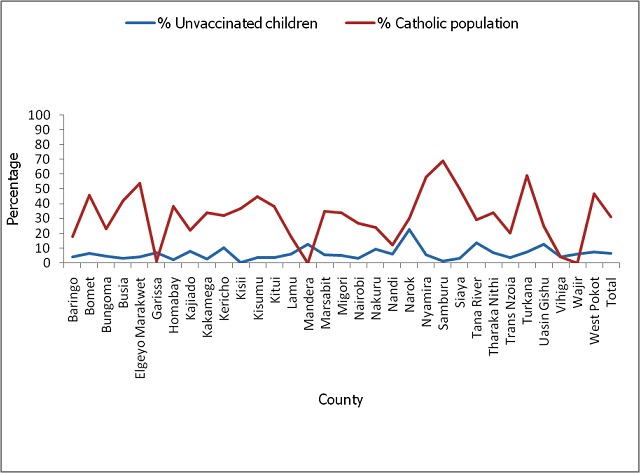
Comparison of unvaccinated children with the catholic population, August 2015

**Figure 2:**
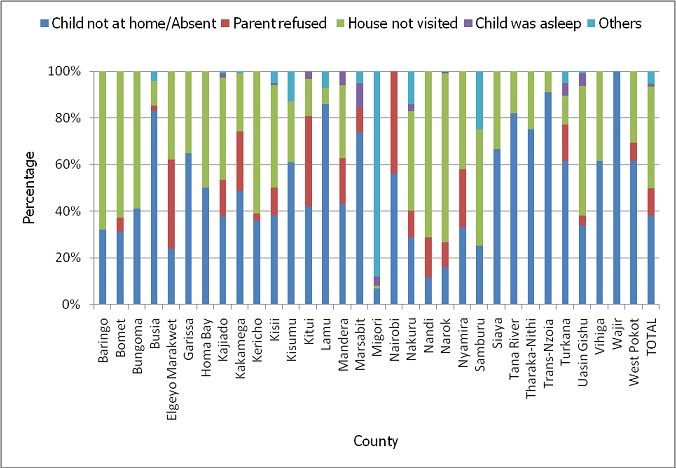
Reasons for non vaccination during the August 2015 polio campaign

**Figure 3:**
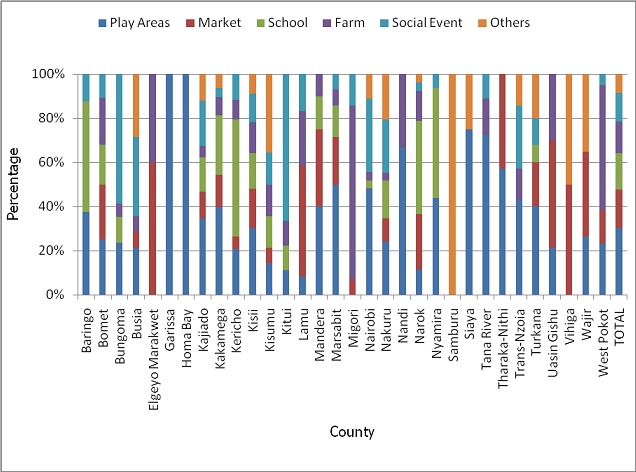
Reasons for child absence during the August 2015 polio campaign

A total of 90,157 children were sampled in the August 2015 post polio campaign survey that was done in 32 counties compared to 163,056 children that were sampled in 47 counties during the November 2014 post campaign survey. The national survey coverage for the August 2015 campaign was 93% while that of November 2014 was 94%. This represented a small decline of 1% but which was statistically significant (p value <0.0001). However, the coverage varied by county in all the 32 counties that conducted the campaign in August 2015 with 13 (41%) counties having statistically significant decline in performance compared to November 2014 campaign. Eleven (34%) counties had statistically significant increase in performance while 8(25%) had no significant change compared to the November 2014 campaign (Table 1). A total of 37,732 parents/guardians of targeted children were sampled during the August 2015 post campaign survey. Seventy two percent of them accepted to answer the question on religion of which 31% belonged to Catholic religion, 50% to Protestant religions, 15% to Muslim religion while 4% belonged to other minority religions. Figure 1 shows the proportion of parents/guardians that were Catholic compared to the proportion of children that were not vaccinated in each county. Overall, 31% of the sampled parents/guardians were Catholic but only 7% of the children were not vaccinated. Various reasons contributed to the 7% of the children that were not vaccinated in the August 2015 campaign. Overall, 44% of the children who were not vaccinated were missed because their houses were not visited by the vaccinators. Another 38% were missed because they were not at home at the time of the visit, 12% because their parents declined vaccination, 1% because the children were asleep while 5% were missed due to various other reasons. However, the reasons for non vaccination varied by county as shown in Figure 2. Compared to the November 2014 campaign, the proportion of children who were not vaccinated due to parents refusal significantly increased from 6% in November 2014 to 12% in August 2015 (p-value< 0.0001). A number of reasons were given for children being away from home at the time of visit by the vaccinators during the August 2015 campaign. Although this varied by county as shown in Figure 3, overall 30% of the children were missed because they were playing outside, 18% because they were in the market, 16% because they were in school, 14% because they were in the farm, 13% because they were attending a social event and 9% due to other reasons.

**Table 1:** Comparison of Polio Vaccination Coverage between November 2014 and August 2015

		November 2014			August 2015			
	County	Children Assessed	Children Finger marked	% Vaccinated	Children Assessed	Children Finger marked	% Vaccinated	P value	Change
1	Baringo	3015	2748	91	2045	1967	96	<0.0001	Increased
2	Bomet	2263	2178	96	2987	2792	93	<0.0001	Decreased
3	Bungoma	4354	4186	96	3753	3580	95	0.03	Decreased
4	Busia	4095	3963	97	2257	2183	97	NS	No Change
5	Elgeyo Marakwet	1054	1038	98	1786	1715	96	0.0002	Decreased
6	Garissa	6219	5883	95	3281	3050	93	0.0013	Decreased
7	Homabay	6649	6465	97	3533	3454	98	NS	No Change
8	Kajiado	3853	3604	94	4699	4333	92	0.018	Decreased
9	Kakamega	9523	9078	95	3929	3818	97	<0.0001	Increased
10	Kericho	2787	2721	98	1762	1582	90	<0.0001	Decreased
11	Kisii	5282	5225	99	2575	2566	100	0.0008	Increased
12	Kisumu	3742	3569	95	2401	2319	97	0.02	Increased
13	Kitui	1383	1272	92	3012	2900	96	<0.0001	Increased
14	Lamu	759	646	85	1092	1028	94	<0.0001	Increased
15	Mandera	3253	2970	91	2088	1821	87	<0.0001	Decreased
16	Marsabit	131	128	98	942	892	95	NS	No Change
17	Migori	4025	3917	97	3456	3285	95	<0.0001	Decreased
18	Nairobi	24944	24015	96	5913	5731	97	0.0159	Increased
19	Nakuru	7407	6742	91	5189	4702	91	NS	No Change
20	Nandi	2452	2383	97	2323	2190	94	<0.0001	Decreased
21	Narok	2764	2697	98	5558	4292	77	<0.0001	Decreased
22	Nyamira	1659	1549	93	2058	1944	94	NS	No Change
23	Samburu	731	720	98	727	717	99	NS	No Change
24	Siaya	3202	2987	93	2394	2321	97	<0.0001	Increased
25	Tana River	425	419	99	1115	966	87	<0.0001	Decreased
26	Tharaka Nithi	1482	1370	92	1619	1508	93	NS	No Change
27	Trans Nzoia	5681	5161	91	2322	2235	96	<0.0001	Increased
28	Turkana	5765	4830	84	4446	4129	93	<0.0001	Increased
29	Uasin Gishu	2607	2337	90	2050	1795	88	0.0259	Decreased
30	Vihiga	2845	2740	96	3643	3497	96	NS	No Change
31	Wajir	6545	6253	96	2489	2342	94	0.0045	Decreased
32	West Pokot	3375	2902	86	2713	2515	93	<0.0001	Increased
	Total	163056	153762	94	90157	84169	93	<0.0001	Decreased

NS: Not Significant

## Discussion

The vaccination coverage for the August 2015 polio vaccination campaign that targeted 6 million children less than 5 years was 93%. This was a slight drop from the 94% that was achieved during the last campaign in November 2014. Although this drop was small, it was statistically significant (p value <0.0001). The proportion of the population that belongs to the Catholic Church in Kenya as per the last census of 2009 is 23% (31). The proportion of the parents and guardians that belonged to the Catholic Church as per the survey was 31%. This difference between the proportion of the population that belongs to the Catholic by census and by survey (23% versus 31%) could be explained by the fact that only 72% of the sampled parents and guardians accepted to answer the question on religion and it could be possible that some people from other religion did not find it necessary to reveal their religion. The difference could also be explained by the fact that 23% is from census data yet our survey was conducted only in 32 counties that participated in the campaign and there could be religious differences across the country. The coverage survey results shows that only 7% of the targeted children were not vaccinated. This is a relatively small number compared with the 23% of the population that is expected to be Catholic. The drop of only 1% from the previous campaign is also relatively small compared to the proportion of the population that belongs to the Catholic Church. As per the World Health Organization standards, a vaccination campaign is regarded as successful if less than 10% of the children are missed during the vaccination [32]. Therefore the coverage of 93% that was achieved could be regarded as largely successful even if 4 (12.5%) counties had a coverage of less than 90%. The results are also in line with the expected regional and sub regional coverage. For example, of all the polio vaccination campaigns that were done in the African region in 2010, 2011 and 2012, only 7.94%, 6.86% and 5.95% of the children were missed, respectively. During the same years, the proportion of children that were missed in the South and East Africa sub region were 12.4%, 11.26%, 13.69%, respectively [32].

Analysis of the results by county indicated that 13 (41%) counties had statistically significant decline in performance compared to November 2014 campaign. However, further analysis of the county results did not establish any relationship between the decline in performance and the proportion of the population that was Catholic in the counties. The reasons for missed children varied by county but generally religion were not a major contributing factor. This is because of the 7% that was missed, it was mostly because their homesteads were not visited by the vaccinators (44%) or the children were not at home at the time of the visit (36%). Only 12% were missed because their parents declined vaccination for various reasons. Though this was a small proportion, it had increased significantly from 6% in November 2014(p value <0.0001) and it therefore means that the call for boycott had negatively influenced a number of parents not to accept vaccination of their children. Based on the above findings, the call for boycott of the vaccination campaign by the Catholic bishops in Kenya can therefore be regarded as largely unsuccessful. This in contrast to a similar call for boycott of the polio vaccination by the Muslim leaders in Northern Nigeria in 2003 that led to a complete boycott of the campaigns in 3 northern states of Kano, Kaduna, and Zamfara for close to a year. Initially 8 states had objected to the campaign (Kano, Kaduna, Zamfara Bauchi, Jigawa, Katsina, Kebbi, Sokoto) but after consultation with the federal ministry of Health, five of the states accepted to take part in the campaigns [33]. This boycott led to an increase in the number of wild polio cases in Nigeria that also led to international spread to 20 previously polio free countries by 2006 [34].

In both Kenya and Nigeria, the main reason for boycott was that the polio vaccine was contaminated with anti-fertility agents (estrogens). However, in Nigeria, there were also claims that the vaccine contained HIV and cancerous agents [35]. So why did the boycott succeed in Nigeria but fail in Kenya? The reason is that although none of the 2 countries had real evidence for their claims, the backgrounds to the claims were completely different in the 2 countries. In Nigeria, many reasons could have contributed to the success of the boycott. One is that traditionally, there had been low intake of orthodox health services in northern Nigeria compared to the south. For example, Jegede in his article on why Nigeria boycotted the polio campaign writes that utilization of orthodox health services in the northern Nigeria was 18% in 1990, 11% in 1999 and 8% in 2003 compared to 50%, 60% and 64% in southern Nigeria, respectively. He further adds that the government of Nigeria in the 1980s had introduced a policy of not more than 4 children per woman and that part of the community believed that the immunization campaigns was one of the strategies to achieve this target [35]. Another major reason for the boycott in northern Nigeria is that there were already suspicions about western health interventions following the unsuccessful trial of a new meningococcal meningitis antibiotic called trovan (trovafloxacin) in 1996. This trial to test the new drug was sponsored by Pfizer in Kano (one of the 3 states that boycotted the polio campaign) during a meningitis outbreak. The community claimed that Pfizer (a multinational drug maker) with the endorsement of federal Ministry of Health and UN bodies used Nigerians in Kano as guinea pigs to test a new unapproved drug without ethical approval [33, 35]. This controversy continued for many years and contributed to the boycott. Political differences between the North and South could also have contributed to the success of the boycott in northern Nigeria. The country had just conducted a presidential election in May 2003 and the opposition led northern states had opposed the election of the federal government which was seen to be southern led. The opposition to the polio campaign could therefore be regarded as disapproval of the policies of the southern led federal government that had just been elected [35]. Unlike the background situation in Nigeria that led to the polio vaccination boycott, the situation in Kenya was different. The major reason for boycott in Kenya was related to the tetanus vaccine controversy in 2014. The Catholic bishops had opposed the vaccination campaign on the argument that the tetanus vaccine had been laced with an anti-fertility drug, Human Chorionic Gonadotrophin (HCG) [25]. Though this issue was resolved after joint testing of vaccines, the bishops had demanded that testing of all vaccines used for future mass vaccination must be done. This is the reason that they demanded the testing of polio vaccines [29]. Even though the government accepted to test the vaccines, there was a disagreement on the methodology to be used and therefore the bishops called for a total boycott of the campaign [30].

The controversy in Nigeria was resolved in 2004. However, there are still cases of refusals during polio vaccination campaigns in northern Nigeria. Reasons often quoted for the refusals include no felt need for the vaccine, vaccine not helpful, lack of trust in government, vaccine not safe and there being more important needs than the polio vaccine [36, 37]. In Kenya, the controversy was resolved through dialogue and testing of the polio vaccine which indicated that the vaccine was safe. Therefore, the Catholic bishops did not call for boycott during the subsequent September and December 2015 polio vaccination campaigns. However, more consultations with the bishops and other stakeholders are needed in order to resolve the differences once and for all in order to avoid a long term negative effect like in Nigeria. This study had a few limitations. First is that although we included a question on the reasons for refusing vaccination, the number of responses was too small for any meaningful interpretation. Second, we did not collect any information as to why some parents/guardians decided to defy the call by the Catholic bishops and instead decided to have their children vaccinated. Third, the study did not collect any information on the strategies that the bishops used to ensure that their call for boycott was successful. Fourth, we also did not collect any information on the strategies that the government used in their response to counter the boycott. We believe that the strategies used by the government to counter the boycott together with the lessons learnt should be documented to help in dealing with similar situations in the future.

## Conclusion

The polio eradication goal of 2018 is now more feasible than ever before because only 2 countries in the world remain endemic (Pakistan and Afghanistan), a 50% reduction from the 4 countries that were endemic 5 years ago. Compared to 2014, the number of wild polio virus cases in 2015 also reduced by more than 80%. However, the gain made so far can easily be reversed by calls for vaccination boycott as was recently made by the Catholic bishops in Kenya in 2015. An analysis of the vaccination coverage revealed that the call for boycott did not affect the campaign significantly. However, there was a slight increase in the number of parents and guardians who refused their children to be vaccinated compared to other campaigns in the past. Therefore, if this call for boycott is repeated in future it could have some significant negative implication to polio eradication as well as other vaccination programs in the country. It is therefore important to ensure that any vaccine safety issues are addressed accordingly.

### What is known about this topic

- Polio is a serious disease that has been earmarked for eradication since 1988;
- Polio eradication milestones have adversely been affected in the past by resistance to vaccination based on religious reasons;
- Significant progress has now been made in the reduction of polio cases globally and eradication is now feasible by 2018.

### What this study adds

- This study provides crucial feedback on the outcome of a polio vaccination campaign following a call for nationwide boycott by the Catholic bishops in Kenya in 2015;
- The results of the assessment shows that many parents did not heed the call for boycott;
- The study gives hope to the polio eradication initiative that has invested a lot in polio eradication
